# Compliance with AAPM Practice Guideline 1.a: CT Protocol Management and Review — from the perspective of a university hospital

**DOI:** 10.1120/jacmp.v16i2.5023

**Published:** 2015-03-08

**Authors:** Timothy P. Szczykutowicz, Robert K. Bour, Myron Pozniak, Frank N. Ranallo

**Affiliations:** ^1^ Department of Radiology University of Wisconsin Madison School of Medicine and Public Health Madison; ^2^ Department of Medical Physics University of Wisconsin Madison School of Medicine and Public Health Madison WI USA

**Keywords:** CT, CT dose, protocol optimization, protocol management, compliance

## Abstract

The purpose of this paper is to describe our experience with the AAPM Medical Physics Practice Guideline 1.a: “CT Protocol Management and Review Practice Guideline”. Specifically, we will share how our institution's quality management system addresses the suggestions within the AAPM practice report. We feel this paper is needed as it was beyond the scope of the AAPM practice guideline to provide specific details on fulfilling individual guidelines. Our hope is that other institutions will be able to emulate some of our practices and that this article would encourage other types of centers (e.g., community hospitals) to share their methodology for approaching CT protocol optimization and quality control. Our institution had a functioning CT protocol optimization process, albeit informal, since we began using CT. Recently, we made our protocol development and validation process compliant with a number of the ISO 9001:2008 clauses and this required us to formalize the roles of the members of our CT protocol optimization team. We rely heavily on PACS‐based IT solutions for acquiring radiologist feedback on the performance of our CT protocols and the performance of our CT scanners in terms of dose (scanner output) and the function of the automatic tube current modulation. Specific details on our quality management system covering both quality control and ongoing optimization have been provided. The roles of each CT protocol team member have been defined, and the critical role that IT solutions provides for the management of files and the monitoring of CT protocols has been reviewed. In addition, the invaluable role management provides by being a champion for the project has been explained; lack of a project champion will mitigate the efforts of a CT protocol optimization team. Meeting the guidelines set forth in the AAPM practice guideline was not inherently difficult, but did, in our case, require the cooperation of radiologists, technologists, physicists, IT, administrative staff, and hospital management. Some of the IT solutions presented in this paper are novel and currently unique to our institution.

PACS number: 87.57.Q

## I. INTRODUCTION

The CT literature has frequently proposed methods with which to reduce radiation dose levels and improve image quality.^1^, ^2^ However, due to the nature of academic publishing, little to no guidance is given on how to best apply the findings to a particular scanner. Work by McKinney et al.^(3)^ does describe a method to propagate CT protocols between vendors and among different platforms from a single vendor. More work of this nature is needed. Even when different versions of a protocol are published for different platforms,^(4)^ the methodology behind the differences in the protocols is not presented. The preliminary work done by our group and others has demonstrated that information technology (IT) can play a key role in allowing for hundreds or thousands of patients' scan data to be used to validate CT protocol optimization^5^, ^6^, ^7^, ^8^ across a wide range of CT platforms. The methodologies currently being developed for protocol propagation are needed to address the growing desire of institutions with a wide variety of CT scanners (e.g., it is common to see centers spanning the spectrum from 8 slice to state‐of‐the‐art 128–320 slice CT scanners) to propagate protocol changes across their entire CT install base. There are multiple commercially available tools which can facilitate protocol optimization and review (i.e., dose monitoring and tracking tools). The future of CT protocol optimization and review using these tools will likely involve the convergence of basic science and big data as more metrics are being developed which can be applied directly to CT images and provide details on facets of CT images related to radiation risk or image quality (e.g., organ dose,^(9)^ image noise,^(10)^ and patient positioning^(11)^).

Having an optimized set of protocols across each scanner at a given institution still does not ensure quality patient care. In reality, these protocols must be properly applied, managed, and reviewed in the clinical environment. The literature is sparse (see the work of Kofer^(12)^ for one example of an exception to this statement) when it comes to proper management techniques for CT protocols and proper methods to review protocols for compliance with ACR mandates.^(13)^ Recently, the practice guideline produced by the AAPM titled “CT Protocol Management and Review Practice Guideline”^(14)^ serves as a minimum standard which clinical physicists can use to gauge how their CT protocol management, review, and optimization program should be run. Our institution recently began to overhaul our CT protocol optimization process concurrently with the release of the AAPM report. One of the results of this effort was our compliance with a majority of the ISO 9001:2008 standards.^(15)^ We propose that following many of the ISO 9001:2008 clauses are beneficial to an institution's CT protocol optimization effort. Individuals not familiar with ISO compliant quality management systems are encouraged to see the work of Biazzo and Bernardi.^(16)^

The methods section of this article will cover each of the sections outlined in the AAPM guidelines.^(14)^ Specifically, we will provide details on how our institution: 1) documents changes to protocols using corrective and preventative action forms; 2) defines what a CT protocol is composed of; 3) defines the roles of the members of our CT protocol optimization team; and 4) manages, reviews, optimizes, and creates new CT protocols with an emphasis on the importance of IT solutions for monitoring protocol performance.

## II. MATERIALS AND METHODS

### A. Documentation: CAPA forms

**Figure A1:**
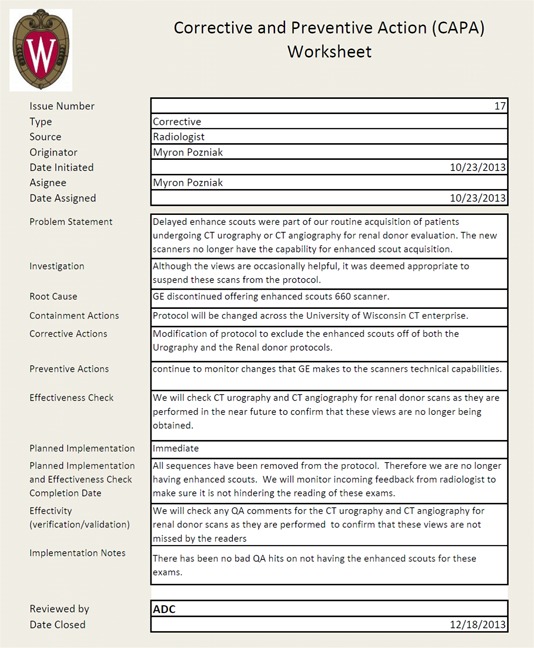
A typical CAPA form. This form is used to document every protocol change. Email communications are frequently attached to these forms, as well as validation and dose data.

The activities detailed in the following sections are documented using Corrective and Preventative Action (CAPA) forms. These forms satisfy the ISO 9001:2008 documentation clauses for routine activities carried out within our quality management system. Our CAPA forms also serve the purpose of assigning tasks to individuals. This provides a tangible means to track the progress of action items. Appendix Fig. A1 depicts a typical CAPA form filled out and archived for future reference at our institution. In practice these forms are easy to fill out. We frequently attach email communications to these forms to help document discussions. In addition, when relevant, we attach dose (i.e., ${\text{CTDI}}_{\text{vol}}$ or DLP) data or radiologist feedback on image quality.

We use CAPA forms to document changes to all of the pieces of our protocols (defined in Materials & Methods section B). In addition to protocol changes, we also document format changes to our protocol documents with CAPA forms. Changes to daily and weekly quality assurance testing protocols and procedures are also documented with CAPA forms. Lastly, changes to clinician education materials detailing the use of our QA system (described in Materials & Methods section D.2) are also documented using CAPA forms.

For institutions not interested in documenting protocol changes in this detail, we urge them to look at their protocols and ask questions like, “Why was this protocol changed two years ago?” or “Who authorized this change?” Such questions, in our experience, are usually difficult or impossible to answer, especially after personnel changes, and are usually difficult even for the people who made the original changes.

### B. Definition of a protocol

The AAPM practice guidelines define a CT protocol as the “collection of settings and parameters that fully describe a CT examination.”^14^, ^17^ Being a university research and teaching hospital, we have been exposed to a wide range of CT protocols. We have seen others' “CT protocols” range from having protocols reside solely as acquisition parameters saved on a CT scanner console to detailed instructions covering many aspects of the scan including patient preparation, positioning, scan acquisition parameters, and image reformat instructions.

CT protocols at our institution (see Appendix B1, B2) are composed of multiple sections. A design philosophy description provides our medical personnel with guidance on when to use a given protocol and any special considerations that should be taken into account. A clinical instructions section is composed of patient preparation instructions, patient positioning instructions, contrast injection parameters (injection rates, amount of contrast and saline chaser, power injector program selection details), and whether or not the scan should be monitored by a radiologist. Localizer (i.e., scout, topogram, scanogram, or surview) scanning instructions and technique factors are listed. Technique parameters for each phase of the exam including reconstruction and reformat options are listed. Lastly, our protocols contain PACS networking instructions.

### C. Team member roles

The following section lists the percent effort and specific job duties unique to the CT protocol optimization team at our institution and only the percent effort related to protocol optimization and management. The duties we describe below should not be taken as the only way to divide responsibilities within a CT protocol optimization team, they merely reflect the current implementation of our quality management system at the University of Wisconsin Madison. We currently have ten diagnostic CT scanners, three PET/CT systems, and two radiation oncology CT scanners on site, and are responsible for eight community CT scanners. Each of our diagnostic (non‐PET/CT and nonradiation therapy) scanners have roughly 300 protocols.

#### C.1 Lead CT radiologist (20% FTE)

The lead CT radiologist is responsible for coordinating efforts between all aspects of the CT protocol optimization team. Most importantly, this position is responsible for “ensuring the promotion of awareness of customer requirements throughout the organization” (ISO clause 5.5.2^(15)^). Without individual radiologists accepting the result of the CT protocol optimization team's efforts (e.g., by not requesting changes to the scan acquisition parameters at scan time), consistent image quality cannot be obtained. As most individuals have a preference on performing an exam a certain way, which is often backed up by studies from the literature, individual institutions must decide on a uniform way to perform studies. When CT technologists are asked to change protocols depending on who is the current attending radiologist in the reading room, mistakes can be made. The lead CT radiologist at our institution is instrumental in maintaining a culture of open discussion of protocol changes within — and sometimes between — sections and in ensuring everyone is familiar with the benefits of protocol uniformity.

The lead CT radiologist also ensures the other radiologists are familiar with the CAPA documentation system, outlined in the Materials & Methods section A. of this article. These forms are completed by our radiologists before any protocol revisions are made. This ensures protocol changes are not made “on the spot” by a radiologist at the scanner. Our CT technologists are also educated to not allow such changes. This is facilitated by limiting the number of individuals with authority to modify protocols on a scanner. In order to comply with the ISO standard for document control (ISO clause 4.2.3^(15)^), our lead CT radiologist must sign protocol release authorization forms before protocol changes are made active on a scanner.

The lead CT radiologist is also responsible for ensuring other radiologists receive training in our quality assurance system, outlined in Materials & Methods section D.2. While our project manager keeps records of radiologist education in our QA system, it is sometimes necessary that individual radiologists are reminded of the importance of completing these activities from one of their peers.

Lastly, the lead CT radiologist is responsible for prioritizing the efforts of the entire CT protocol optimization team. At any given moment, we have around six different protocol revisions in progress. Determining what order these tasks should be completed is the task of the lead CT radiologist.

#### C.2 Section CT lead radiologist (5% FTE for five positions)

Each section lead CT radiologist is responsible for reviewing the quality assurance data with the CT protocol optimization team. The interval for these reviews varies at our institution, but for routine protocols these reviews occur at least four times per year. For new protocols, or protocols with new revisions, quality assurance data are reviewed immediately upon the use of the new or modified protocol. Section lead radiologists are also responsible for distilling the wishes of their section and communicating these with the CT protocol optimization team. The section lead radiologists also must settle disagreements on alternate scanning techniques to ensure uniformity of protocols within their section.

In order to comply with the ISO standard for document control (ISO clause 4.2.3^(15)^), section lead CT radiologists must sign protocol release authorization forms before protocol changes are made active on a scanner for protocols within their section.

#### C.3 CT physics (100% FTE)

Physics is responsible for making changes to CT protocol acquisition and reconstruction parameters. Changes can be due to optimizing a current protocol, adapting protocols to new scanners, or creating new protocols. At our institution, protocol changes are motivated by and guided by data we collect in the quality assurance IT solution we describe in Materials & Methods section D.2. Physics is also responsible for educating radiologists and CT technologists on the function of the various scan options of each scanner. This education usually takes the form of relating the differences in image quality and any logistical characteristics like total exam time and or tube heating limits. Specifically, physics will reconstruct clinical and or phantom images using different settings to educate the clinical staff. Changes in total exam time can easily be computed and such numbers are also presented to the clinical staff. Physics also assists in protocol revision and checks all protocol parameters within our protocol documents against what is actually programmed into the scanner. In addition, physics is responsible for monitoring the percent acceptance of each protocol, something which is a critical activity for any CT imaging center, in our opinion. One of our physics staff received ISO internal auditor training and is our ISO representative when we are audited for ISO compliance.

In order to comply with the ISO standard for document control (ISO clause 4.2.3^(15)^), our lead CT physicist or supporting physicist must sign protocol release authorization forms before protocol changes are made active on a scanner.

#### C.4 Lead CT technologist (40% FTE)

The lead CT technologist is responsible for notifying the other CT technologists of changes to the protocols, maintaining updated electronic copies of protocols at each CT scanner, entering protocol changes and new protocols onto the scanner, notifying billing and coding personnel when changes need to be made, notifying the IT personnel who maintain our clinical CT protocol system when changes have to be made and, most importantly, providing “logistical” information to the rest of the CT protocol optimization team. By logistical we refer to information pertaining to: realistic patient breath hold times for different age patients, realistic patient breath hold times for patients with different clinical indications, and realistic maximum tube mA values (senior CT technologists have the best idea of what mA values can routinely be used without causing the CT scanner to have long delays for tube‐cooling or limited maximum mA values for subsequent scans).

Our lead CT technologist is also responsible for properly creating and storing the CAPA forms, explained in Materials & Methods section A. In addition, our lead CT technologist is responsible for controlling revisions within our actual CT protocol documents and keeping track of the numbering of our protocols. We maintain uniform numbering for all protocols across all of our scanners. Uniformity in naming and numbering^(12)^ of protocols across all of their scanners is important as this reduces the chance of error as CT technologists move from scanner to scanner.

In order to comply with the ISO standard for document control (ISO clause 4.2.3^(15)^), our lead CT technologist must sign protocol release authorization forms before protocol changes are made active on a scanner.

#### C.5 Project manager (15% FTE)

The CT protocol optimization team manager is responsible for organizing team meetings and taking minutes, keeping action item lists current, and assisting in grammatical and formatting protocol revisions.

#### C.6 Quality management and ISO 9001:2008 consultant (50% FTE for six months)

In order to set up our quality management system and ensure we were complaint with ISO 9001:2008, we hired a part‐time credentialed quality consultant.

Upon arriving, our quality consultant interviewed each CT protocol optimization team member. Once the consultant understood what motivates a protocol change, who needs to authorize the change, who implements the change, and who checks to ensure the change was implemented correctly, and finally that our radiologists approve of the new resultant images, our consultant came up with a documentation system to record each of these steps. The description of each step makes up a piece of our quality management system, the enforcement and documentation of each step is what the ISO 9001:2008 clauses provide. The description of how each facet of our quality management system is enforced is located in procedure documents broken up according to each ISO clause.^(15)^

#### C.7 Quality management fellow (20% FTE)

We currently have an abdominal imaging fellow on our CT protocol optimization team. This individual assists in monitoring the percent acceptance rate of each of our protocols across each of our scanners and body sizes. In addition, our quality fellow edits our quality management system documents, as well as our ISO procedure documents.

#### C.8 IT support (7% FTE)

We currently house our quality management document, ISO procedures, CAPA forms, protocols, training materials, and other miscellaneous forms on an instance of SharePoint (Microsoft Corporation, Redmond, WA). The upkeep of this program requires ongoing support from our radiology's department IT group.

The quality assurance data collection system, described in the Materials & Methods section D.2, was written entirely in‐house by our supporting physicist and two IT specialists. Upkeep for the database storing the results from the system, as well as ongoing changes to the graphical user interface used to capture radiologist feedback, is the responsibility of our radiology's IT department.

### D. Managing, reviewing, and optimizing protocols

#### D.1 Managing protocols

Paper copies of our CT protocols are not used at the scanner. Instead, our technologists use Microsoft Word (Microsoft Corporation, Redmond, WA) and Adobe Acrobat Pro (Adobe Systems Inc., San Jose, CA) on computers directly adjacent to our CT scanner consoles. All of the components described in the Materials & Methods section B are available to our technologists.

Our protocol documents are written such that they are applicable to all of the scanners within our CT install base. In other words, we do not have to create separate documents for each scanner. We feel this is an important aspect of our protocol management system because it saves our CT protocol optimization team time and reduces the chance for error. This is because while we tune the technical acquisition parameters differently for each scanner, the other protocol components (described in B above) are the same across all of our scanners with only a few exceptions for some of our oldest and newest scanners. Having a single document covering all scanners enables quick and efficient changes in those portions of the CT protocol that are not related to the actual acquisition parameters. Maintaining as many parameters as possible in one location that can be shared between scanners is essential in our experience. The ultimate goal for maintaining a single set of CT protocols across a large install base of CT protocols is uniformity. Uniformity in CT enables radiologists to rule out changes in a patient's anatomy/pathology being caused by differences in technical acquisition factors, patient prep and setup, or contrast injection parameters from previous studies.

The technical acquisition parameters for our scanners are stored in a spreadsheet in which each scanner model has its own column. We also use this format for the reconstruction parameters. See Appendix B1, B2 for an example of our CT protocols. Protocol parameters that are shared between scanners and that are unique for a given scanner are easily observed. All of our reconstruction parameters (slice width, kernel, vendor specific reconstruction options, etc.) are almost exactly the same since we have all GE Healthcare CT scanners (GE Healthcare, Waukesha, WI). This facilitates our protocol layout. Institutions with multiple vendors can use the lexicon developed by the AAPM^(17)^ to relate parameters across vendors in order to use our protocol layout.

To be compliant with ISO 9001:2008 clause 4.3 (Documentation Requirements),^(15)^ we maintain old versions of all protocol documents using SharePoint (Microsoft Corporation). SharePoint ensures only individuals with the proper authorization can edit documents, and allows us to archive old versions of protocols documents so an accurate record of our protocol revision history can be maintained. This program is far superior to simply having a shared network drive in which documents can be saved. We had such a system, but found it difficult to monitor the editing history. SharePoint automatically keeps track of editing history.

#### D.2 Reviewing protocols

Our protocols can be reviewed by one of four pathways: 1) during our annual physics testing, 2) during routine review of quality assurance data, 3) every time a protocol revision is made, and 4) at the urging of or clinicians or technologists.

Our annual physics testing includes measuring the ${\text{CTDI}}_{\text{vol}}$ for those protocols mandated to be checked in order to obtain ACR accreditation. The team reviews all of the scan and reconstruction parameters, validating that the parameters on each scanner match what is in the protocol documents. This annual review only covers 11 out of our ∼300 protocols. Only pediatric/adult head and body protocols are mandated to be checked by the ACR. Our other protocols are largely based on the 11 protocols checked to satisfy the ACR. Therefore, finding errors in the ACR‐mandated protocols automatically triggers a more thorough review. In other words, an error observed in the routine abdominal protocol triggers a review of all other protocols, including a series in which the routine abdomen scan parameters are used.

**Figure 1:**
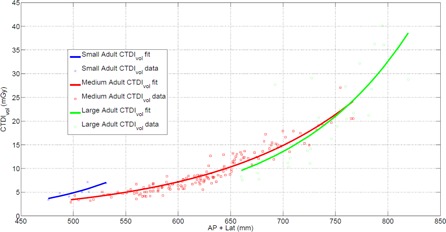
Example plot of the ${\text{CTDI}}_{\text{vol}}$ value as a function of patient size for small, medium, and large versions of the same protocol.

**Figure 2:**
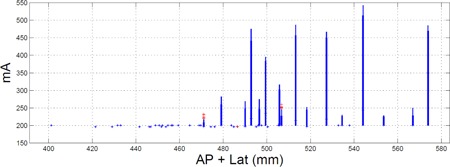
Tube current statistics as a function of patient size. In this example, the mA is clearly “minning” out for smaller patients which suggests the minimum tube current should be lowered. Here the averaged tube current used for each slice within a single series of an exam is plotted as a function of patient size. The plots are box and whisker plots, in which the whiskers extend to all points not considered outliers and the box to the 10th and 90th percentile. The red “plus” symbols denote points calculated to be outliers.

ACR accreditation requires that protocols be reviewed for the following: 1) “Review appropriate settings for patients of various sizes … Ensure that appropriate CTDI values result from these settings before patients are scanned with protocol”; 2) “Review appropriate settings for patients of various sizes … including the noise index, quality reference mAs, and other tube current modulation settings”; and 3) “Protocols should be reviewed for acceptable image quality for the diagnostic task required”.^(13)^ At our institution, we track the ${\text{CTDI}}_{\text{vol}}$ values of our protocols as a function of patient size using custom software that measures the anterior posterior and lateral patient sizes from scout images, as is shown in Fig. 1. Figure 1 depicts the ${\text{CTDI}}_{\text{vol}}$ for a single protocol modified for three different patient sizes — small, medium, and large. Such plots let us understand how the scanner output changes as a function of patient size and between protocols. In order to review the appropriateness of AEC function, we rely on plots of tube output statistics as a function of patient size.^(18)^ Assuming the AEC is not “minning” or “maxing” out the tube current, the resulting image noise and dose should behave in a predictable manner. Here we refer to the mA as “minning”/“maxing” out when it reaches the lower/upper limit of the CT scanner or the lower/upper limit defined by the operator respectively. Figure 2 depicts a plot of tube current statistics for which the minimum mA was set too high, as is obvious from the “minning” out of the mA distributions at small patient size. (Note: comparing 2, 4 makes it easy to appreciate how the distribution in Fig. 2 is “minned” out for small patient sizes.) When the tube current is “minned” out, the scan time can likely be decreased and or the dose can be decreased. Plots, like those shown in Fig. 2, can also be useful for identifying protocols which are “maxing” out the tube, a more common occurrence in our experience. However, such “minning” and “maxing” out can be avoided if the methodology outlined in the Materials & Methods section D.3 is followed.

**Figure 3:**
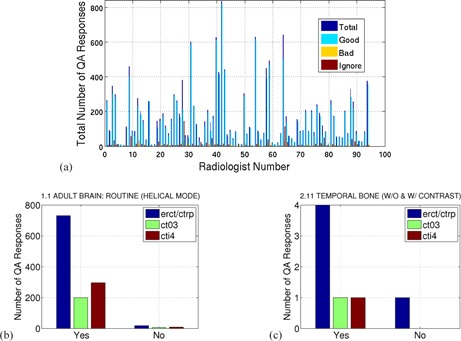
Graph (a) detailing the overall participation of individual radiologists with our quality assurance system. “Ignore” denotes the radiologists choosing not to answer either “good” or “bad”. Detailed summary (b) of “auto QA” data for a particular protocol over a particular date range broken up by scanner model demonstrating good acceptance for a routine head protocol. Detailed summary (c) of “auto QA” data for a particular protocol over a particular date range broken up by scanner model demonstrating poor acceptance for one scanner and low numbers of exams for all scanners for a temporal bone protocol. “erct/ctrp” refers to a GE LightSpeed VCT scanner, “ct03” refers to a GE Optima 660 CT scanner, and “cti4” refers to a GE HD Discovery CT scanner.

We satisfy the ACR requirement for acceptable image quality review due to the implementation of a quality assurance system which captures radiologist feedback on every CT exam read by our radiologists and residents. To date, we have over 37,000 responses in our database.^7^, ^8^ Our system is composed of two levels of data collection. The first system we refer to as “auto QA” and presents our radiologists with a simple binary decision for every exam they read. The radiologist has the choice to respond that the exam was either “good” or “bad” and when “bad” they have the opportunity to leave a textual comment. Our “auto QA” system enjoys 100% participation by our radiologists (attendings and fellows) and residents. Figure 3 depicts our overall clinician participation in our “auto QA” system and a couple examples of the type of information we can get from system. The second level of data collection we refer to as “exam QA”. “Exam QA” is where more detailed comments can be entered and where complaints concerning improper scanning methods and improper image reformats are made. We have created training material for our radiologists and residents describing each of these systems and training records are maintained following ISO standards. There is an important difference between our “auto QA” and “exam QA” systems. The former provides an idea of how well a given protocol is performing and does not account for errors at scan time or in the creation and sending to PACS of images. Technologists' errors or issues with image quality due to PACS display problems do not count against protocol performance in our system. This granulation in exam acceptance is critical to obtain a robust volume of data which can be used to monitor the performance of protocols on our scanners.

We maintain a database that houses “auto QA” responses, protocol name, scanner model, date, clinician performing the “auto QA”, dose information (CTDI_vol_ and DLP), patient position within the scan FOV (measured from the scout images), and patient size (measured from the scout images^(19)^) linked using the exam accession number. Quarterly reviews of the “bad” responses are conducted for each section with the section lead radiologist, CT lead radiologist, and a physicist. Details about each reviewed exam are recorded and the validity of the “bad” response is determined. We only review “bad” responses for protocols with fewer than ten total QA responses or for protocols with less than a 95% acceptance (“good”/(“good” + “bad”)) rate. Each time a protocol revision is made, the totals for “good” and bad” responses are reset. To date, we have rejected 60% of the “bad” responses in our database as being made in error. A majority of our “bad” responses that are rejected are classified as “misclicks”, meaning the section lead radiologist felt the image quality was adequate but the “bad” button was mistakenly hit by the reviewing radiologist. We also reject “bad” responses for issues relating to the presence of artifacts and patient motion; both of these factors are usually not due to the protocol and should be noted using a different QA tool available to our radiologists.

The review notes are maintained in a disposition log. We analyze the log each quarter to identify radiologists requiring guidance on how to use the quality assurance system. In our experience, the review of protocols in this fashion is quite efficient at quickly discovering issues with protocols on specific scanners. Analyzing our “auto QA” data has generated some research projects as correlations among “bad” responses are easy to see. For example, we realized during a review session that every “bad” response for our routine small adult abdomen pelvis exam was positioned too low in the gantry. This led us to perform a study in which we concluded the optimal positioning for these types of exams (abdomen pelvis) was not the geometric center of the patient but the “center of mass” which positions the patient's spine closer to isocenter. This reduces image noise nonuniformity and reduces beam hardening artifact.^(20)^

The last avenue motivating a protocol change is when a clinician or technologist contacts the CT protocol team directly. A recent example of this type of change was the addition of a delayed phase to our hepatocellular carcinoma liver protocol in order for our protocol to conform to the United Network for Organ Sharing (UNOS) guidelines. A recent technologist motivated change was to raise the smartprep (GE Healthcare's name for contrast bolus monitoring; see the AAPM lexicon for other vendors^(17)^) mA settings in order to allow better visualization of the aorta on large patients. Our current quality assurance feedback system currently does not support technologist responses, so direct feedback is required.

Our quality management system dictates that every time a protocol is changed, it is reviewed by that section's lead radiologist, physics, and the lead CT technologist. Before a new protocol (or newly changed protocol) can be used clinically on the scanner, it is entered by a physicist or our lead CT technologist onto the scanner. Then the entry of the protocol is double‐checked to ensure it was entered correctly by a person other than the one who initially entered it. This process is aided at our institution by the presence of stand‐alone CT independent consoles (ICs). Protocols can be entered and checked on these ICs before they are used clinically. For scanner models for which we do not have ICs, before changes are made, a copy of the current protocols is made so the original protocols can be loaded back onto the scanner after the protocol revisions have been made. This is done for those cases in which the check cannot be performed immediately after the new changes are made. The revised protocols are saved to a disc and are only used clinically after being checked (which can be done after hours, between patients when the throughput is low, or on weekends). Sometimes we check the protocols at entry by having the “checker” watch the person doing the entering. Many scanners offer outputs of protocols in spreadsheet formats, which can also be used to check protocols off‐line. In addition, we have successfully read protocol files (.proto files extensions for GE Healthcare) directly from our scanner's operating system using in‐house written software. Yet we do not recommend this approach due to the proprietary nature of protocol formats which can change without notice from the vendor.

#### D.3 Optimizing protocols

We have around 300 protocols spanning our radiology department's neuro, abdominal, pediatric, thoracic, cardiovascular, and musculoskeletal sections. For each body region, we identified protocols that we refer to as “basis protocols”. Using these “basis protocols”, different dose levels can easily be made by changing the noise level on the automatic exposure control (noise index for GE, quality reference mAs for Siemens, mAs per slice for Philips, standard deviation for Toshiba and Hitachi). However, changes to more than the noise level are often required, to minimize dose or provide diagnostically useful images to our clinicians. Therefore changes in kV, pitch, slice thickness, and so on must also be made. The two most important factors in determining if a given set of parameters will work on a given scanner is the scan duration and the ability of the scanner to provide the required tube current.^(18)^

In addition to making protocol changes in dose or scan time on a single scanner, we propagate protocol changes from one scanner to another. For scanners with relatively low maximum mA limits the scan time (i.e., total period of beam‐on time) must be prolonged to provide enough output (e.g., by decreasing the pitch or increasing the tube rotation time). Often, scanners with relatively low maximum mA limits are 8‐ or 16‐slice scanners. When compared to 64 or larger slice scanners, they suffer longer scan times due to their smaller available beam width. We use mathematical relationships to quantify how changes in scan acquisition parameters affect the required tube current and to bring some quantification to the “art” of balancing so many factors. An example of such a relationship is shown in Eq. (1):

(1) $$mA_{new}=mA_{basis}\frac{T_{basis}P_{new}}{T_{new}P_{basis}}{\left(\frac{NI_{basis}^{*}}{NI_{new}^{*}}\right)}^{2}F_{kV}F_{D}$$

In Eq. (1), “basis” refers to the scanner from which a protocol is being translated from (the basis scanner can be equal to the new scanner for protocol changes on a single scanner), *T* is the tube rotation time, *P* is the pitch, NI* is the noise index (see the AAPM lexicon^(17)^ for the analogous automatic exposure control parameter for other vendors) normalized to a nominal slice thickness, $F_{W}$ takes into account changes in tissue contrast due to changes in beam energy, and $F_{D}$ takes into account changes in image noise due to the presence or absence of denoising algorithms. Other F factors can be used to account for more complicated differences between scanners. For example, geometric differences that affect scanner output can be accounted for. We have successfully implemented this method for translating a single protocol to different dose levels, beam energies, slice thicknesses, and/or scan times within a single scanner. We have also used this methodology to propagate protocols between 64‐slice and older 32‐ and 16‐slice scanners.

**Figure 4:**
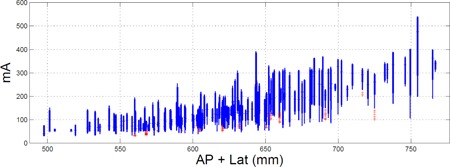
Example plot used to obtain reference minimum and maximum mA values. Here the averaged tube current used for each slice within a single series of an exam is plotted as a function of patient size. The plots are box and whisker plots in which the whiskers extend to all points not considered outliers and the box to the 10th and 90th percentile. The red “plus” symbols denote points calculated to be outliers.

In practice, Eq. (1) is used to ensure the tube current limits of a scanner are not exceeded when changing variables like rotation time, noise level, slice thickness, beam energy, or pitch. Figure 4 depicts the distribution of mA values as a function of patient size for one of our abdomen pelvis protocols. Using such plots, realistic values for the maximum and minimum tube current can be obtained for various patient sizes. If ${\text{mA}}_{\text{new}}$ is discovered to be over the maximum mA limit of the scanner (or under the minimum limit), Eq. (1) provides a framework to study how changes in acquisition parameters will affect the tube current used by the AEC. For a given scanner, up‐front work including experimental data collection is needed to quantify the F parameters listed in Eq. (1), but in some cases these values can be analytically calculated or taken from the literature (e.g., the effect of beam energy on iodine contrast has been well published). Further details will be provided in a future publication.

It should be noted that the above analysis has worked quite well for us in practice, even though the AEC control has been shown to vary for different sized patients between scanner models of the same manufacturer.^(3)^ Since we only propagate protocols between the same anatomical regions and for similar sized patients, differences in AEC function due to large changes in patient size do not have a large influence on our method.

## III. DISCUSSION & CONCLUSION

It was the aim of this article to provide an overview of the duties of the CT protocol optimization team and how it specifically addresses the minimum practice guidelines outlined by the AAPM in practice guideline 1.a.^(14)^ We hope this article will help institutions understand the amount of effort required to manage, review, and optimize CT protocols. We also hope this article will allow comparing and contrasting between other centers who currently have robust CT protocol optimization teams in place and ourselves, so as a community we can share our experiences. It is likely that our approach to complying with the AAPM's minimum guidelines would not be feasible for all institutions. It is also likely other similar institutions (university research hospitals with on‐site medical physics support) would do things differently. It is likely that small community centers or large centers without IT and physics support could not implement many of the methods we have discussed in this paper. For these centers we suggest following the basic ACR guidelines for annual protocol review using dose data provided by the ACR dose index registry and anecdotal information concerning protocol performance to guide protocol changes (or encourage your radiologists to keep a list of exams they liked and disliked throughout the year). Most small centers will not have hundreds of CT protocols and therefore less time should be required for CT protocol optimization. In addition, the AAPM is currently releasing protocols for a wide range of scanner makes and models; those centers who see optimizing their own protocols as a daunting task should take advantage of these AAPM protocols.

In closing, it should be noted that even for centers with IT departments, on‐site medical physicists, technologists, and radiologists willing to work on their protocols; without top level management support efforts to manage, review, and optimize CT protocols will be hampered. A significant amount of time is required for these tasks, as outlined in Material & Methods section C. Management needs to recognize this and ensure the members of the CT protocol team have sufficient time resources. Unfortunately, the cost of optimizing protocols due to this large percent time effort by members of the CT protocol optimization team is not trivial.^(21)^ Management also has to enforce the uniformity required by any quality management system. Individual radiologists must be willing to forgo having technologists alter protocols for their own unique preferences.

## ACKNOWLEDGMENTS

The authors would like to thank the members of the UW Madison CT protocol optimization team: Amanda Ciano, Richard Bruce, Lisa Aumann, Pete Wasmund, Gary Wendt, Jeff Kanne, Scott Nagle, Kara Gill, Mike Hartman, Daryn Belden, Ken Schreibman, and Pam Ziemlewicz. We are grateful to Meredith Albrecht for editorial assistance. The author's institution receives an equipment grant from GE Healthcare. Additionally, the authors supply CT protocols to GE Healthcare under a licensing agreement; Myron Pozniak is lead investigator in this effort.

## Figures and Tables

**Figure B1:**
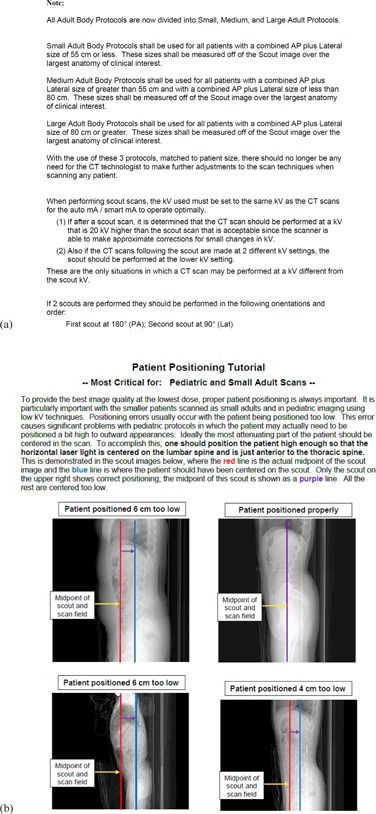
Patient sizing instructions (a) for our CT technologists; patient positioning tutorial (b) for our CT technologists.

**Figure B2:**
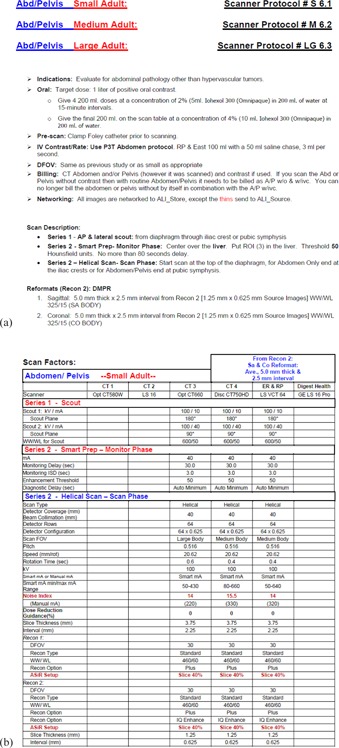
An example UW CT protocol. This is a routine abdomen pelvis protocol which is read primarily by our abdominal imaging section. Scanning instruction (a) specific to a single protocol (e.g., contrast information, reformat information). Technical acquisition and reconstruction parameters (b) for a single protocol (this particular protocol has two more pages for medium and large adults, only the small adult technical parameters are shown). Additionally, we have “design philosophy” documents for each section describing the clinical indications motivating the use of a particular protocol (not shown).
